# Targeting pyroptosis for skeletal muscle atrophy: mechanistic insights and therapeutic perspectives

**DOI:** 10.3389/fimmu.2026.1896679

**Published:** 2026-08-24

**Authors:** Chen-Chen Sun, Tian-Jiao Peng, Peng Zhang, Xin-Long Fan, Chong-Tao Lu, Hui-Ling Jiang, Chang-Fa Tang

**Affiliations:** 1 School of Physical Education, Hunan First Normal University, Changsha, Hunan, China; 2 Laboratory of Immunotranslational Research, Translational Immunology in Traditional Chinese Medicine, College of Pharmacy and Food, Southwest Minzu University, Chengdu, Sichuan, China; 3 Key Laboratory of Physical Fitness and Exercise Rehabilitation of the Hunan Province, College of Physical Education, Hunan Normal University, Changsha, Hunan, China

**Keywords:** Gasdermin D, NLRP3 inflammasome, pyroptosis, skeletal muscle atrophy, therapeutic target

## Abstract

Skeletal muscle atrophy is a progressive syndrome characterised by the loss of skeletal muscle mass and function. It significantly impairs patients’ quality of life and clinical prognosis. Current intervention strategies have limited efficacy, necessitating the identification of novel therapeutic targets. Pyroptosis, a form of programmed cell death mediated by the Gasdermin protein family and accompanied by intense inflammatory responses, has been found to play a pivotal role in skeletal muscle homeostasis. Through core signalling axes NLRP3-caspase-1-GSDMD, it triggers the release of large amounts of inflammatory cytokines, establishing and sustaining a chronic inflammatory microenvironment that drives protein degradation. This review systematically elucidates the core molecular mechanisms of pyroptosis, revealing its role in exacerbating skeletal muscle atrophy through direct damage to myofibres and the establishment of chronic inflammatory microenvironments. It summarises potential intervention strategies targeting pyroptosis, further analysing key challenges in current research and prospects for clinical translation.

## 1 Introduction

Skeletal muscle, as the largest metabolic and locomotor organ, accounts for approximately 40% of adult body weight and plays a central role in maintaining energy homeostasis and motor function (1). Its structural and functional integrity is vulnerable to impairment, leading to skeletal muscle atrophy characterised by progressive loss of muscle mass and strength, which severely impacts patients’ independence and clinical prognosis (2). Skeletal muscle atrophy arises from diverse causes, encompassing age-related sarcopenia, cancer cachexia, and various chronic wasting diseases, and has become an increasingly significant public health burden (3, 4). This pathological muscle wasting directly compromises patients’ motor function and is associated with an increased risk of complications and mortality (5). Whilst a few targeted drugs have emerged with limited efficacy, current clinical management still predominantly relies on nutritional support and rehabilitation exercise. Consequently, elucidating novel mechanisms and exploring novel therapeutic strategies have become urgent in this field.

Pyroptosis, a form of programmed cell death mediated by the Gasdermin protein family (represented by GSDMD), has gained attention for its pivotal role in linking innate immunity with tissue homeostasis regulation (6). A hallmark of the classical pyroptotic cascade is the activation of inflammatory caspases (such as caspase-1, -4, -5, and -11). Once activated, these proteases cleave Gasdermin family members, liberating the N-terminal domain responsible for pore formation. The subsequent integration of these domains into the plasma membrane disrupts osmotic balance, culminating in cell swelling, lysis, and the consequent efflux of substantial quantities of pro-inflammatory cytokines (7). Initially identified in anti-infective immunity, pyroptosis is now recognised as extensively involved in the pathological processes of various chronic non-infectious diseases (8). Notably, recent research indicates that pyroptosis is involved in regulating skeletal muscle homeostasis. Its activation is closely associated with NLRP3 inflammasome assembly, cytokine release, and the progression of muscle atrophy (9, 10). Abnormal expression and activation of key pyroptosis molecules have been observed across animal models of skeletal muscle atrophy induced by diverse aetiologies (11–14). This suggests that pyroptosis constitutes a pivotal mechanism underlying multiple muscle-wasting disorders.

This review aims to systematically elucidate the specific activation mechanisms and functional roles of pyroptosis in various types of skeletal muscle atrophy, comprehensively analysing the pathways through which it drives the pathological network of muscle atrophy. Through a systematic examination of the pyroptosis molecular network and its complex regulatory role in muscle atrophy, to provide a theoretical foundation and novel therapeutic perspectives for developing novel strategies targeting pyroptosis regulation in the prevention and treatment of muscular disorders.

## 2 Pyroptosis

### 2.1 The discovery process and characteristics of pyroptosis

A landmark breakthrough occurred in pyroptosis in 2015, when Shao Feng’s team identified Gasdermin D (GSDMD) as the core effector protein (15, 16). Research indicates that inflammatory caspases (caspase-1/4/5/11) cleave GSDMD, releasing its N-terminal domain to form pores in the cell membrane. This leads to cell swelling, lysis, and the release of inflammatory mediators. This pore-forming mechanism, directly mediated by the Gasdermin protein, constitutes the most fundamental molecular characteristic of pyroptosis and represents its essential distinction from apoptosis (**Table 1**). Subsequently, the functions of other members were progressively elucidated, revealing crossovers with the apoptotic pathway: for instance, the apoptotic executor caspase-3 cleaves GSDME under specific conditions, thereby redirecting the process towards pyroptosis. This provides a plausible explanation for the potent inflammatory side effects associated with certain chemotherapeutic agents (17). In 2020, research further identified a novel pyroptosis pathway triggered by lymphocyte toxin granzyme B, directly cleaving gasdermin proteins (GSDMB/E), expanding understanding of their role in adaptive immunity (18).

**Table 1:** Compared the key characteristics of apoptosis and pyroptosis.

Types	Core properties	Morphological characteristics	Key executive factor	inflammatory response	Detection marker
Apoptosis	Non-inflammatory programmed cell death	Cellular shrinkage, chromatin condensation, formation of apoptotic bodies, maintenance of membrane integrity	Caspase-3/6/7, Bcl-2 family	Weak or non-inflammatory	Caspase-3 activation, Annexin V binding, TUNEL positivity
Pyroptosis	Promoting pro-inflammatory programmed cell death	Cell swelling, membrane permeabilisation, and release of cellular contents	Gasdermin protein family (GSDMD/E.)	Severe inflammatory response (release of IL-1β/IL-18)	GSDMD-N-terminal processing, LDH release, IL-1β maturation

### 2.2 Core molecular mechanisms of pyroptosis

#### 2.2.1 Core executor: pore-forming effect of GSDM protein

**Figure 1:**
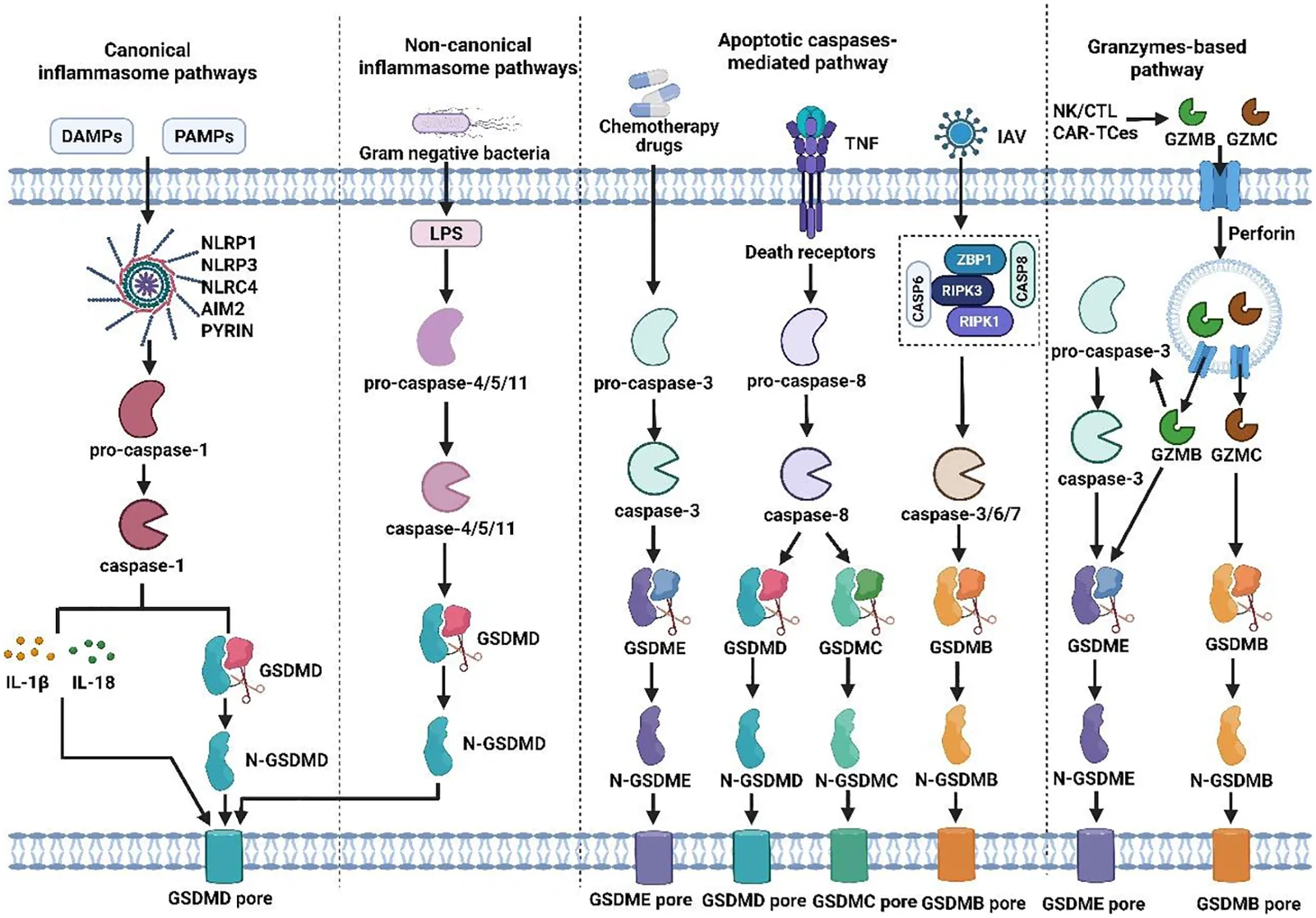
Mechanisms of pathways involved in pyroptosis. (1) Canonical inflammasome pathways: DAMPs and PAMPs activate sensors such as NLRP1, NLRP3, NLRC4, AIM2, and PYRIN, leading to pro-caspase-1 cleavage into active caspase-1. Caspase-1 then processes IL-1β and IL-18 and cleaves GSDMD to form pores in the membrane. (2) Non-canonical inflammasome pathway: Intracellular LPS from Gram-negative bacteria directly activates pro-caspase-4/5/11, which cleaves GSDMD to induce pore formation. (3) Apoptotic caspases-mediated pathway: Chemotherapy drugs, TNF, and Influenza A Virus (IAV) activate ZBP1, RIPK3, and RIPK1, leading to caspase-8 activation and subsequent cleavage of caspase-3/6/7. These caspases cleave GSDME, GSDMD, GSDMC, and GSDMB, contributing to membrane permeabilisation. (4) Granzyme-based pathway: NK cells, CTLs, or CAR-T cells release granzymes (e.g., GZMB, GZMC) that activate caspase-3 or directly cleave gasdermins, leading to pore formation and pyroptosis.

Currently, four primary signalling pathways are involved in recognising and inducing pyroptosis, including the canonical and non-canonical inflammasome pathways, the apoptotic caspase-mediated pathway, and the granzyme-based pathway (**Figure 1**) (19). Although the pathways differ, the core mechanism relies on the pore-forming effect of the GSDM protein family on cell membranes. The family comprises six members: GSDMA, GSDMB, GSDMC, GSDMD, GSDME, and PJVK/DFNB59 (20). Except for PJVK/DFNB59, the others comprise the N-terminal pore-forming domain (NTD) and the C-terminal repressor domain (CTD). As the ultimate effector molecule of pyroptosis, GSDM activity is strictly regulated: in the resting state, its NTD is self-inhibited by CTD. When danger signals are present, upstream proteases specifically cleave GSDMD, releasing the NTD and oligomerizing. This forms pores approximately 10–15 nanometres in diameter across the cell membrane, leading to cell swelling and lysis (21, 22). This constitutes the critical structural basis for pyroptosis execution.

The upstream proteases cleaving GSDM proteins primarily include inflammatory caspases and apoptotic caspases. Caspase-1 is activated in the classical pyroptotic pathway, forming an inflammasome multiprotein complex, whereas caspase-4/5/11 (human/mouse) belongs to the non-canonical pathway, directly recognising and binding cytoplasmic lipopolysaccharide (LPS) without an inflammasome complex (23, 24). Both of the inflammatory caspases cleave GSDMD, triggering its pore formation. Apoptotic caspases (caspase-3/8) cleave specific GSDM family members (GSDME, GSDMD/C) under certain conditions (exposure to certain chemotherapeutic agents), redirecting cellular fate from non-inflammatory apoptosis towards inflammatory pyroptosis (17, 25). This reveals intricate interactions and fate conversion mechanisms within the programmed cell death network.

#### 2.2.2 The classical inflammasome pathway

The core mechanism of pyroptosis is highly dependent upon activation of the classical inflammasome pathway. This pathway serves as the central hub for the host to recognise danger signals and initiate programmed inflammatory cell death, comprising intracellular pattern recognition receptors (PRRs), apoptosis-associated speck-like protein containing a caspase-recruitment domain (ASC), and inflammatory caspases. PRRs, acting as inflammasome detectors, recognise pathogen-associated molecular patterns and danger-associated molecular patterns (26). The prevalent PRRs include NLRP1, NLRP3, NLRC4, absent in melanoma 2, and Pyrin (27). NLRP3 perceives multiple endogenous danger signals, including extracellular ATP, reactive oxygen species (ROS), and mitochondrial DNA; NLRP1 is activated by specific microbial products; NLRC4 collaborates with NAIPs to recognise bacterial flagellar proteins; AIM2 directly binds cytoplasmic double-stranded DNA; whilst Pyrin recognises host protein modification induced by bacterial toxins (27).

**Figure 2:**
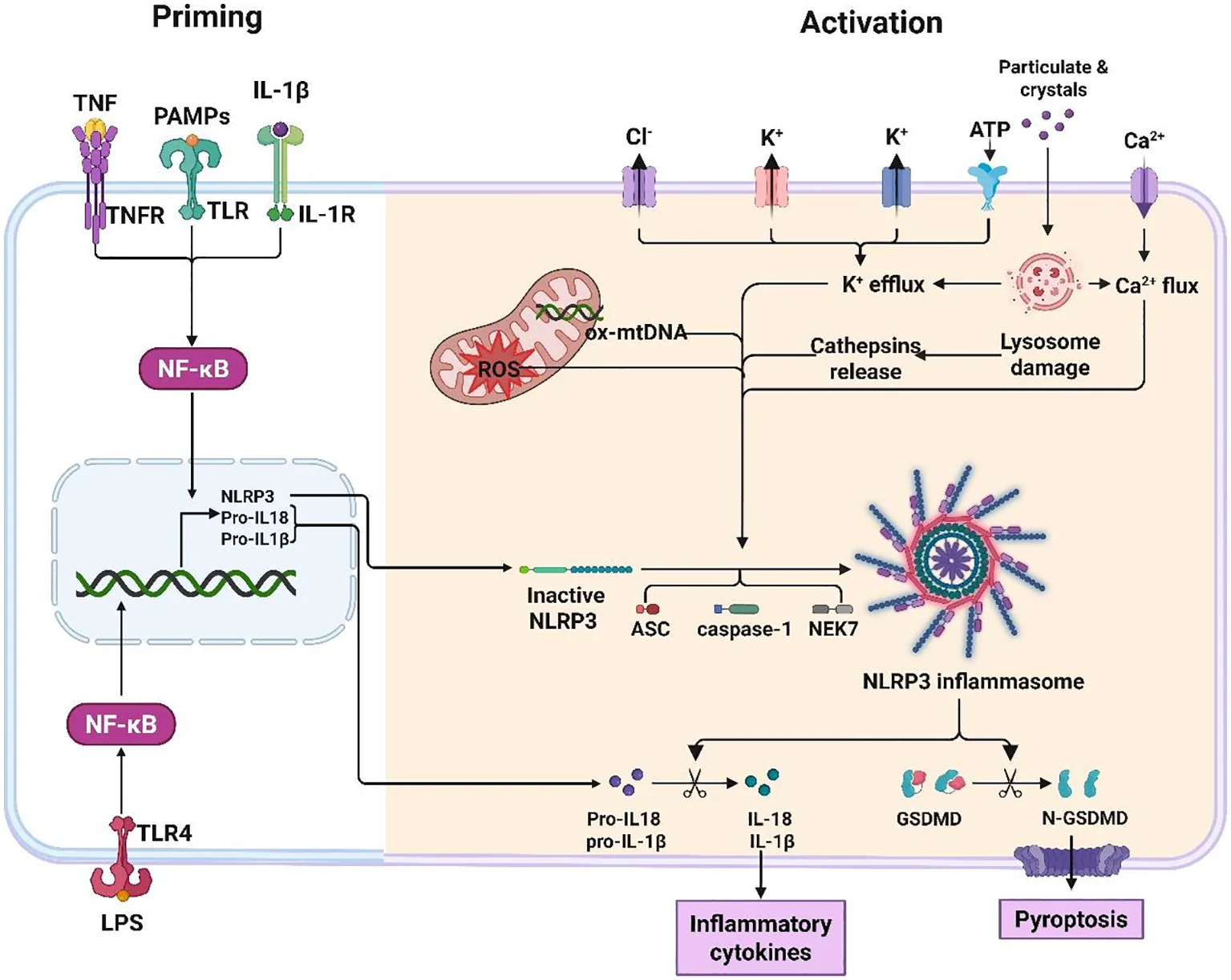
Molecular mechanism of NLRP3 inflammasome activation. The NLRP3 inflammasome is activated through sequential priming and triggering signals. During the priming phase, PAMPs, TNF, or IL-1β engage TLRs or the IL-1 receptor (IL-1R), activating NF-κB signalling to upregulate the expression of NLRP3, pro-IL-1β, and pro-IL-18. In the subsequent activation phase, diverse stimuli—including ATP, particulate matter/crystals, and lysosomal damage—trigger cellular stress signals such as K^+^ efflux, Ca^2+^ flux, ROS production, and cathepsin release. These signals promote NLRP3 inflammasome assembly with the adaptor protein ASC, NEK7, and pro-caspase-1. The activated caspase-1 then cleaves pro-IL-1β and pro-IL-18 into mature inflammatory cytokines, whilst also cleaving GSDMD to generate its N-terminal fragment. N-GSDMD forms membrane pores, leading to pyroptotic cell death and the release of mature cytokines.

Upon recognising ligands, PRRs recruit ASC via their domains and activate pro-caspase-1. Activated caspase-1 cleaves GSDMD and processes pro-IL-1β and pro-IL-18 into mature forms for release (**Figure 2**) (28). In skeletal muscle atrophy, inflammatory cytokines released by NLRP3 inflammasome activation create a chronic inflammatory microenvironment, upregulating atrophy-associated genes and exacerbating protein degradation and muscle wasting (29–31). Recent studies indicate that specific inhibition of NLRP3 or knockdown of GSDMD effectively mitigates myotubular pyroptosis and muscle wasting (13).

#### 2.2.3 Non-canonical inflammasome pathways

Within the regulatory network of pyroptosis, the non-canonical inflammasome pathway constitutes an activation mechanism separate from the classical pathway. The core feature is that inflammatory caspases (human caspase-4/5, mouse caspase-11) directly recognise intracellular ligands. During Gram-negative bacterial infection, LPS enters the host cytoplasm via specific mechanisms (such as bacterial secretion systems or lysosomal rupture). It directly binds to the N-terminal CARD domains of caspase-4/5/11, inducing oligomerisation and autoactivation (28). Activated caspase-4/5/11 subsequently cleaves the pyroptosis effector GSDMD, releasing NTD, and forms pores in the cell membrane, driving permeabilisation and cell lysis. Notably, distinct from caspase-1 in the classical pathway, caspase-4/5/11 lacks the capacity to process pro-inflammatory cytokine precursors (pro-IL-1β/IL-18) (32). Additionally, the expression patterns and regulatory mechanisms of NLRP3, GSDMD, and upstream signalling components may differ between species, potentially affecting the translation of rodent findings to human pathophysiology.

The non-canonical pathway amplifies inflammatory responses through crosstalk with the canonical pathway. Its key mechanism involves K^+^ efflux triggered by GSDMD pore formation, as a “second signal” to activate the NLRP3 inflammasome (33). Furthermore, studies reveal that activated caspase-11 cleaves the membrane channel protein Pannexin-1, leading to ATP release; extracellular ATP exacerbates K^+^ efflux by activating its receptor P2X7, positively promoting NLRP3 activation. Activated NLRP3 inflammasome recruits and activates caspase-1, which completes the maturation and release of pro-IL-1β/IL-18 (34). Thus, the non-canonical pathway activates caspase-4/5/11 to cleave GSDMD, forming membrane pores that subsequently induce K^+^ efflux and ATP release. This effectively triggers the activation of the canonical NLRP3-caspase-1 inflammasome pathway, leading to intense inflammatory cell death. This pathway has significant pathological relevance in systemic infections such as sepsis and its potential complications involving muscle wasting.

#### 2.2.4 Apoptotic caspases-mediated pathway

Recent studies have revealed that apoptosis-associated caspases drive pyroptosis under specific conditions, demonstrating the complexity and plasticity of the cell death network. Caspase-3, a core apoptosis executor, has been found to cleave GSDME (35). In cells overexpressing GSDME (certain tumour cells), chemotherapy-induced caspase-3 activation cleaves GSDME, generating its pore-forming N-terminal fragment and converting apoptosis to pyroptosis (25, 36). This mechanism plays a significant role in the inflammatory side effects induced by chemotherapy, offering a novel perspective on tumour immune regulation.

Similarly, caspase-8, a key initiator protease in the apoptosis pathway, was demonstrated as an activator of pyroptosis. It exhibits substrate diversity: in Yersinia infection, its effector protein YopJ induces caspase-8 cleavage of GSDMD by inhibiting TAK1 kinase, thereby triggering pyroptosis (37). In the tumour microenvironment, nuclear-localised PD-L1 and STAT3 upregulate GSDMC transcription; under TNF-α stimulation, caspase-8 is activated and specifically cleaves GSDMC, inducing pyroptosis in tumour cells. Furthermore, research reveals that caspase-3/6/7 cleaves GSDMB (38). These findings establish Caspase-3/8 as a ‘molecular switch’: they determine cellular fate towards non-inflammatory apoptosis or inflammatory pyroptosis based on cell type, stimulus context, and the GSDMs expression profile. Therefore, the apoptosis and pyroptosis pathways are not isolated; they are closely interconnected and mechanistically integrated through GSDME and GSDMC.

#### 2.2.5 Granzyme-based pathway

Apart from the caspase family, granzymes (GZM) secreted by immune cells constitute another crucial pathway for inducing pyroptosis in target cells, playing a pivotal role in tumour immune surveillance. Cytotoxic T lymphocytes (CTLs), natural killer (NK) cells, and chimeric antigen receptor T (CAR-T) cells deliver GZMs into target cells via perforin (18, 39, 40). GZMB, as a key effector molecule, directly cleaves GSDME at specific sites, releasing NTD (41). This mechanism provides novel insights for overcoming tumour cell resistance to caspase-dependent apoptosis.

GZMA has been demonstrated to specifically cleave GSDMB to induce pyroptosis, providing novel insights for tumour immunotherapy (39). GSDMB is highly expressed in multiple epithelial tissues and tumours, making the GZMA/GSDMB axis a crucial defence mechanism for immune cells to eliminate specific tumour cells (41). These GZMs-mediated pyroptotic pathways trigger inflammatory cell death in tumour cells, directly eliminating targets and amplifying inflammatory responses within the tumour microenvironment. This process recruits and activates additional immune cells, thereby establishing a positive feedback cycle for anti-tumour immunity.

## 3 The role of pyroptosis in skeletal muscle atrophy

### 3.1 Pyroptosis and sarcopenia

With the acceleration of global population ageing, age-related skeletal muscle atrophy (sarcopenia) has emerged as a significant public health challenge (42). Its development involves multiple factors, including age-related insulin resistance, reduced satellite cell numbers, intramuscular fat infiltration, protein homeostasis imbalance, chronic inflammation, oxidative damage, and mitochondrial dysfunction (43).

In recent years, pyroptosis has gained attention for its potential involvement in sarcopenia. Studies have demonstrated that pyroptosis is one of the key pathways driving muscle loss and inflammation in sarcopenia (44). Adipose tissue infiltration in aged skeletal muscle elevates systemic inflammation levels, upregulating key pyroptosis-associated factors, thereby promoting myofibrillar loss (45, 46). Animal studies indicate that the expression of NLRP3, GSDMD, Caspase-1, and IL-1β is higher in aged SD rat skeletal muscle. Specific knockout of *NLRP3* or *GSDMD* alleviates myotubular atrophy by regulating protein synthesis and degradation pathways and inhibiting the caspase-1-mediated pyroptosis pathway (47). Furthermore, NLRP3 deficiency markedly improved mitochondrial structural damage, alleviated interstitial fibrosis and apoptosis in aged mice, indicating a pivotal role for NLRP3 in muscle degenerative changes and atrophy (48, 49).

The accumulation of mitochondrial damage and excessive ROS during muscle ageing constitutes a pivotal upstream event triggering pyroptosis. ROS, acting as potent injurious signals, directly activate the NLRP3 inflammasome. This subsequently initiates caspase-1 cleavage of GSDMD and the maturation of IL-1β/IL-18, thereby completing the activation of the classical pyroptotic pathway (30, 50). This directly induces inflammatory death of muscle fibres, and released inflammatory cytokines establish a self-amplifying chronic inflammatory cycle, persistently driving muscle catabolism. Therefore, targeting the NLRP3-caspase-1-GSDMD axis of pyroptosis provides a new strategy for intervening in sarcopenia.

However, emerging evidence suggests that pyroptosis is not exclusively detrimental in skeletal muscle physiology. Under physiological conditions, pyroptosis supports immune surveillance (51). Cao et al. demonstrated that GSDME-mediated pyroptotic signalling promotes muscle regeneration via IL-18 signalling (52). *Gsdme* deficiency delayed functional recovery and exacerbated injury-induced myosteatosis, whilst exogenous IL-18 reversed these defects in aged muscle. This finding establishes the GSDME–IL-18–TRM axis as a metabolic checkpoint that sustains lipid homeostasis to support regeneration, revealing a beneficial facet of pyroptosis under physiological or controlled conditions. Thus, excessive activation of the NLRP3–caspase-1–GSDMD pathway drives chronic inflammation and accelerates atrophy, whereas low-level, spatially confined GSDME-mediated pyroptosis participates in muscle repair and regeneration.

### 3.2 Pyroptosis and cancer cachexia

Cancer cachexia is a chronic wasting disease characterised by progressive, irreversible skeletal muscle wasting, accompanied by fat tissue reduction and systemic inflammatory response. According to clinical progression, it is categorised into three stages: pre-cachectic phase (metabolic abnormalities present without significant weight loss), cachectic phase (weight loss >5% within 6 months), and refractory cachexia (unresponsive to anticancer treatment with shortened life expectancy) (53). This syndrome exhibits higher prevalence in invasive tumours such as pancreatic, oesophageal, and lung cancers (54). Notably, recent research indicates muscle wasting may occur months before clinical diagnosis of pancreatic cancer, suggesting that cachexia is not merely a late-stage manifestation but may serve as an early biological hallmark of certain malignancies (55).

Recent researches indicate that pyroptosis plays a significant role in the development and progression of cancer cachexia. The tumour microenvironment and systemic inflammatory responses lead to a significant increase in pro-inflammatory factors. These factors disrupt skeletal muscle protein homeostasis through activation of the ubiquitin-proteasome system and inhibition of the mTOR synthesis pathway, and simultaneously promote the activation of the pyroptosis pathway (56, 57). In mouse cachexia models, activation of the IL-6/STAT3 signalling pathway inhibits muscle protein synthesis, and upregulation of NLRP3 inflammasomes correlates directly with skeletal muscle atrophy (58, 59). These suggest that the pyroptosis pathway may serve as a crucial molecular link between tumour progression and skeletal muscle wasting. Current research has established an intrinsic link between pyroptosis and skeletal muscle degeneration, but the integrated regulatory network requires further investigation.

### 3.3 Pyroptosis and disuse atrophy

Pyroptosis has gained attention for its role in disuse skeletal muscle atrophy (including immobilisation, weightlessness, and denervation). Research confirms that in denervated muscle atrophy models, NLRP3 inflammasomes are significantly activated. The downstream signalling induces pyroptosis via GSDMD, upregulating MuRF1 and Atrogin-1, activating the ubiquitin-proteasome system, and protein degradation (60). Notably, in muscle-specific NLRP3 knockout mice, pyroptosis-related protein expression was suppressed, and the degree of muscle atrophy was also significantly reduced, providing genetic evidence for NLRP3 in the pathological process (60).

Further research has elucidated the mechanism of pyroptosis in disuse atrophy and its potential for intervention. In a rat model of brachial plexus root avulsion injury (BPRA), Edaravone effectively mitigated neuroinflammation, pyroptosis, and secondary muscle atrophy by inhibiting the NLRP3/GSDMD/caspase-1 axis (61). In the post-stroke sarcopenia model, a broader form of cell death termed PANoptosis (a programme of death simultaneously involving apoptosis, pyroptosis, and necrotic apoptosis) was identified. It was confirmed that the Chrysanthemum extract could effectively alleviate muscle degeneration and restore protein homeostasis by inhibiting the expression of key proteins, including Z-DNA-binding protein 1 (ZBP1) and its downstream GSDMD-N, effectively alleviating muscle degeneration and restoring protein homeostasis (56). These findings suggest that targeting upstream signalling hubs of pyroptosis (NLRP3, ZBP1) or its effector proteins (GSDMD) may be an effective strategy for preventing and treating disuse atrophy. Furthermore, in amyotrophic lateral sclerosis (ALS) patients, plasma levels of miR-223 (a microRNA targeting NLRP3 for inhibition) were significantly reduced, whilst NLRP3 and IL-1β levels were markedly elevated. These alterations showed significant correlation with the severity of functional impairment and decline in respiratory function (62). This suggests that the circulating miR-223/NLRP3/IL-1β axis may serve as a biomarker for disease progression assessment and a potential therapeutic target.

Although targeting pyroptosis provides new therapeutic avenues for treating disuse atrophy, current evidence is predominantly derived from rodent models; the safety profile and the long-term risks associated with inhibitors targeting key pyroptotic effectors such as NLRP3 and GSDMD remain to be established in humans. Furthermore, the dynamic changes in pyroptosis across different stages of muscular atrophy, its interactions with other forms of cell death, and the specific regulatory mechanisms within distinct muscle fibre types require further elucidation.

### 3.4 Pyroptosis and disease-related muscle atrophy

Pyroptosis also plays a significant role in acute metabolic disorders such as diabetes and sepsis. In diabetic muscle atrophy models, STING protein exacerbates pyroptosis by activating NLRP3 inflammasomes, whereas STING deficiency markedly improves muscle function (63). Notably, the NLRP3-specific inhibitor MCC950 independently ameliorates diabetic myopathy and exhibits synergistic protective effects when combined with aerobic exercise (64). Furthermore, bone morphogenetic protein-7 (BMP-7) has been demonstrated to improve diabetic myopathy by inhibiting hyperglycaemia-induced pyroptosis (65). In sepsis-induced muscle wasting, selenomethionine mitigates pyroptosis and inflammation by inhibiting the ROS/NLRP3 signalling axis (30), whilst *Gsdmd* gene knockout significantly alleviates muscle wasting by suppressing the IL-18/AMPK pathway (66). Furthermore, in COPD-associated muscle wasting, the histone deacetylase inhibitor trichostatin A (TSA) alleviates cigarette smoke exposure-induced muscle atrophy by downregulating pyroptosis (67).

These studies confirm that the NLRP3/GSDMD pyroptosis pathway serves as a common pivotal mechanism underlying muscle atrophy in multiple disease conditions. Future research should explore the translational potential of pathway-specific inhibitors (such as MCC950) across different disease models, alongside multimodal strategies combining nutritional interventions (ketogenic diets) and exercise therapy, thereby offering novel approaches for the clinical prevention and treatment of disease-associated muscle wasting.

### 3.5 Pyroptosis and drug-induced skeletal muscle atrophy

Several commonly used clinical drugs may induce skeletal muscle atrophy through activation of the pyroptosis pathway, constituting a serious adverse effect. Glucocorticoids (such as dexamethasone, DEX) represent one of the typical agents inducing drug-induced myopathy (68). Research indicates that DEX treatment significantly upregulates NLRP3, caspase-1, GSDMD, and IL-1β expression in myocytes, activating NLRP3/GSDMD-mediated pyroptosis whilst inhibiting the PI3K/AKT/FoxO3a signalling pathway, ultimately leading to myofibrillar loss (69–71). Notably, trimetazidine effectively mitigates DEX-induced myotubular pyroptosis and atrophy by restoring PI3K/AKT signalling and inhibiting the NLRP3/GSDMD pathway (13). Similarly, the natural product lotus leaf ethanol extract (NNL) and the fucoxanthin derivative significantly alleviate DEX-induced muscle atrophy by downregulating the NLRP3 inflammasome, caspase-1 activation, and GSDMD cleavage, concurrently inhibiting excessive activation of the ubiquitin-proteasome system and autophagy pathways (70, 72).

Apart from DEX, statins (such as simvastatin) activate the NLRP3-caspase-1 axis by inducing ROS accumulation, driving GSDMD-dependent pyroptosis and disrupting autophagic flux, which collectively promote muscle atrophy (73, 74). D-galactose (D-gal), as an ageing inducer, simultaneously upregulates NLRP3 and caspase-11 via mitochondrial damage and oxidative stress, activating classical and non-classical pyroptosis (75, 76). LPS directly activates caspase-4/5/11-mediated non-canonical pyroptosis and sequentially activates the NLRP3 inflammasome via potassium efflux, ultimately upregulating expression of atrophy markers Atrogin-1/MuRF1 (13, 77, 78). These drug-induced pyroptosis pathways ultimately lead to muscle cell membrane perforation mediated by Gasdermin proteins and the release of IL-1β/IL-18, creating a chronic inflammatory microenvironment. This systemically activates the NF-κB pathway to upregulate muscle-specific E3 ubiquitin ligase expression, synergistically promoting protein degradation. Consequently, the mechanism of drug-induced myopathy has expanded to encompass a novel dimension of inflammatory programmed cell death. Targeting key nodes within the pyroptosis pathway may represent a novel therapeutic strategy for preventing and treating drug-related skeletal muscle atrophy.

### 3.6 Muscle fibre subtype-specific susceptibility to pyroptosis

The preceding sections have delineated the role of pyroptosis in various etiologies of skeletal muscle atrophy at the whole-muscle level. However, skeletal muscle is a heterogeneous tissue composed of type I slow oxidative fibres and type II fast glycolytic fibres. Accumulating evidence suggests that type II fibres are more susceptible to NLRP3 inflammasome activation and pyroptosis under various atrophy conditions. The glycolytic metabolism of type II fibres generates less ATP per glucose molecule and produces more metabolic byproducts, creating a cellular environment that exacerbates oxidative stress and mitochondrial dysfunction; both are critical upstream triggers for NLRP3 inflammasome assembly (79). Furthermore, type II fibres exhibit lower expression of endogenous antioxidant enzymes, including superoxide dismutase and glutathione peroxidase, impairing their capacity to neutralise ROS accumulation (80). These properties predispose type II fibres to ROS-mediated NLRP3 activation, caspase-1 cleavage, GSDMD pore formation, and subsequent inflammatory cell death.

In contrast, type I fibres display relative resistance to pyroptotic activation under equivalent atrophic stimuli. Their reliance on oxidative phosphorylation is supported by a dense mitochondrial network and efficient electron transport chain coupling, which limits excessive ROS production under basal conditions (81). Type I fibres possess higher levels of antioxidant defences and more efficient autophagic-lysosomal degradation pathways, facilitating the clearance of damaged organelles and thereby reducing the availability of DAMPs (82). Additionally, the superior capillary density and oxygen supply in type I fibres also promote the rapid removal of metabolic waste and inflammatory mediators, mitigating sustained pro-inflammatory signalling (83). However, whether type I fibres are completely refractory to pyroptosis under severe or prolonged inflammatory stress remains to be determined. Based on the aforementioned mechanisms, the following intervention strategies can alleviate muscle atrophy by targeting the pyroptosis pathway.

## 4 Potential intervention targets for pyroptosis

### 4.1 Non-pharmacological interventions

#### 4.1.1 Exercise intervention

Exercise, as a non-pharmacological intervention, has been extensively employed to improve various chronic diseases and functional impairments, including skeletal muscle atrophy. Recent studies have revealed that inhibiting pyroptosis represents a key common mechanism through which exercise exerts cross-organ protective effects. For instance, exercise-induced circFndc3b promotes Klf2 expression by binding to the ENO1 protein, thereby inhibiting NLRP3 inflammasome-mediated pyroptosis and mitigating cerebral ischaemic injury (84). Moderate-intensity exercise alleviates traumatic brain injury by enhancing mitochondrial autophagy and reducing STING-mediated microglial pyroptosis (85). Regular training suppresses NLRP3/caspase-1/GSDMD pathways activated by oxidative stress, preventing diabetic bone loss (14). Exercise alleviates chondrocyte pyroptosis in osteoarthritis by modulating P2X7/AMPK/mTOR or directly inhibiting NLRP3/GSDME pathways, mitigating various myocardial injuries (86–88). These studies indicate that the protective effects of exercise are closely linked to its inhibition of tissue-specific pyroptosis pathways.

In the prevention and treatment of skeletal muscle atrophy, exercise plays a particularly crucial role in inhibiting pyroptosis. Regular physical activity suppresses NLRP3 inflammasome activation by improving mitochondrial function and reducing the release of endogenous danger ROS (12), downregulating the expression of NLRP3, caspase-1, GSDMD-NT, and IL-1β, mitigates inflammatory cell death in myocytes, enhancing muscle quality and function (47, 64). Notably, exercise and targeted interventions (MCC950) exhibit synergistic effects; their combined application demonstrates superior efficacy to monotherapy in improving muscle fibre morphology and locomotor capacity in diabetic myopathy models (64). This provides a robust theoretical foundation and translational prospects for developing integrated strategies combining exercise prescription with targeted therapies to prevent and treat skeletal muscle atrophy and associated metabolic disorders.

#### 4.1.2 Dietary intervention

Scientific and rational nutritional interventions are key strategies for maintaining health, delaying ageing, and preventing multiple chronic diseases. Within the field of skeletal muscle health, specific nutrients such as protein, vitamin D, n-3 polyunsaturated fatty acids (n-3 PUFAs) from fish oil, and β-hydroxy-β-methylbutyrate have been demonstrated to exert positive effects on maintaining or increasing muscle mass and strength (89). These nutrients provide essential synthetic substrates and exert systemic effects by regulating metabolic and immune pathways.

Recent studies have further revealed that the anti-atrophic effects of certain dietary components are closely linked to their regulation of cell pyroptosis, particularly through inhibiting the NLRP3 inflammasome-mediated classical pyroptosis pathway. For instance, n-3 polyunsaturated fatty acids exert significant anti-inflammatory effects by activating nuclear receptors PPARγ, directly inhibiting NLRP3 inflammasome assembly and activation. This reduces caspase-1-mediated cleavage of GSDMD and the mature release of pro-inflammatory cytokines, ultimately blocking pyroptosis-driven muscle protein degradation (90–92). Short-chain fatty acids alleviate sarcopenia in rats by suppressing NF-κB/NLRP3-mediated inflammation and pyroptosis (93). Vitamin D, besides directly regulating muscle gene expression via the VDR receptor, also demonstrates potential for modulating immunity and alleviating inflammation, potentially indirectly influencing inflammasome activity (94, 95). Rhodiola rosea-derived nanovesicles improve blood flow and attenuate skeletal muscle ischemic atrophy by inhibiting pyroptosis of vascular endothelial cells (96). Furthermore, protein/amino acid supplementation (whey protein and leucine) may indirectly alleviate the inflammatory response by improving the metabolic microenvironment and promoting anabolic processes, with enhanced efficacy when combined with exercise (97). These findings indicate that targeting NLRP3 inflammasome-mediated pyroptosis pathways has emerged as a significant frontier in dietary interventions for preventing and treating muscle atrophy. However, the synergistic interactions between different nutrients and their precise molecular mechanisms require further in-depth investigation.

### 4.2 Pharmacological interventions

#### 4.2.1 Melatonin

Melatonin, an endogenous hormone primarily secreted by the pineal gland, plays a pivotal role in antioxidant defence, anti-inflammatory responses, and maintaining cellular homeostasis (98). Recent studies reveal that melatonin exhibits significant regulatory capacity for pyroptosis, providing a novel mechanistic explanation for its multiple organ-protective effects. For instance, melatonin inhibits doxorubicin-induced myocardial pyroptosis by activating the Sirt1/Nrf2 pathway (99) and mitigates cardiomyocyte pyroptosis during ischaemia-reperfusion injury via the MT2/miR-155/FOXO3a/ARC signalling axis (100). Furthermore, it mitigates microglial pyroptosis by blocking the cGAS-STING-NLRP3 inflammatory cascade (101) and improves stress-induced hippocampal pyroptosis and depressive-like behaviour by inhibiting the Cathepsin B/NLRP3 pathway (102).

In metabolic disorders, melatonin suppresses hyperglycaemia-induced endothelial cell pyroptosis by activating the Nrf2/HO-1 pathway (103) and mitigates macrophage pyroptosis in atherosclerosis by modulating the SIRT3/FOXO3α/ROS axis (104). Notably, following mitochondrial-targeted modification, melatonin exhibits a distinct role of action within the tumour microenvironment, specifically inducing pyroptosis in castration-resistant prostate cancer cells by triggering mitochondrial dysfunction and ROS (105). This bidirectional regulatory capacity demonstrates that melatonin can precisely modulate cellular fate according to microenvironmental differences: suppressing excessive pyroptosis to preserve function in normal tissues, whilst promoting pyroptosis to clear aberrant cells under specific pathological conditions. Melatonin provides novel therapeutic perspectives and potential intervention targets for preventing and treating skeletal muscle atrophy through inhibiting NLRP3 inflammasome activation, regulating oxidative stress balance, and maintaining mitochondrial function.

#### 4.2.2 BMP-7

Bone morphogenetic protein 7 (BMP-7), as a member of the transforming growth factor-β family, plays a crucial role in tissue development and regeneration. Recent studies have revealed that BMP-7 exhibits a novel protective mechanism in various diabetic complications by inhibiting pyroptosis. In diabetic skeletal muscle, hyperglycaemia and lipid accumulation activate the HMGB1/TLR4/NLRP3 inflammasome pathway, inducing caspase-1/GSDMD-dependent myocyte pyroptosis, which leads to muscle atrophy and fibrosis. Notably, BMP-7 intervention significantly ameliorates this pathology and improves muscle structure and function (65, 106). In diabetic cardiomyopathy, BMP-7 improves cardiac remodelling and function by inhibiting TLR4-NLRP3 inflammasome complex activation mediated by Nek7/GSDMD, thereby reducing pyroptosis and inflammatory cell infiltration (107). Furthermore, in diabetes-associated intervertebral disc degeneration, BMP-7 delays degeneration by inhibiting NLRP3 inflammasome activation and pyroptosis in nucleus pulposus cells (108). These cross-organ studies demonstrate that one core protective mechanism of BMP-7 targets the NLRP3 inflammasome-mediated pyroptosis pathway to mitigate inflammatory tissue damage driven by diabetic metabolic disorders. This suggests its potential as a multi-target therapeutic agent for diabetic muscle atrophy and associated complications.

#### 4.2.3 Trimetazidine

Trimetazidine (TMZ) is a clinically established anti-anginal agent. Recent research has revealed its multi-organ protective effects, particularly demonstrating distinct pharmacological actions in inhibiting pyroptosis. This provides novel mechanistic insights for treating skeletal muscle atrophy. TMZ mitigates muscle damage by inhibiting the activation of GSDMD through intervention in distinct signalling pathways. In a glucocorticoid-induced skeletal muscle atrophy model, TMZ downregulates NLRP3/GSDMD-mediated pyroptosis by activating the PI3K/AKT/FoxO3a pathway, thereby mitigating myotube atrophy (13). Furthermore, TMZ reduces cardiomyocyte pyroptosis in myocardial ischaemia/reperfusion injury by inhibiting the TLR4/MyD88/NF-κB/NLRP3 axis (109). In sepsis models, it promotes neutrophil migration and mitigates myocardial pyroptosis via the AMPK/Nrf2/CXCR2 pathway (110). Notably, chiral borneol derivatives designed based on the core structure of TMZ (2,3,4-trimethoxybenzene) exhibit anti-pyroptosis activity in myocardial injury by binding and inhibiting caspase-1, thereby blocking the GSDMD/IL-1β pathway (111). This suggests the structural potential for developing pyroptosis-targeting therapeutics. In summary, TMZ and its derivatives modulate the NLRP3/caspase-1/GSDMD pathway, offering novel targets and therapeutic directions for investigating the mechanisms of skeletal muscle atrophy and developing treatment strategies.

#### 4.2.4 Dieckol

DK is a natural phlorotannin compound extracted from Ecklonia cava, exhibiting anti-inflammatory, antioxidant, and metabolic regulatory physiological activities. Recent studies have revealed that DK exerts protective effects in various metabolic and inflammatory diseases by inhibiting the NLRP3 inflammasome and its downstream pyroptosis pathway. Specifically, in a high-fat diet-induced non-alcoholic fatty liver disease (NAFLD), DK suppressed activation of the HMGB1/TLR4/NF-κB pathway, downregulated expression of the NLRP3/ASC/caspase-1 inflammasome, and reduced pyroptosis, thereby improving hepatic lipid accumulation and tissue damage (112). DK exhibits protective effects in glucocorticoid-induced skeletal muscle atrophy, via inhibiting RAGE/TLR4-mediated NF-κB activation, reducing NLRP3 inflammasome assembly and caspase-1/GSDMD-dependent pyroptosis, thereby decreasing expression of MuRF-1 and atrogin-1 (72).

### 4.3 Pyroptosis inhibitors

#### 4.3.1 MCC950

MCC950 is a small-molecule inhibitor that specifically targets the NLRP3 inflammasome, effectively suppressing downstream caspase-1/GSDMD-mediated pyroptosis by blocking the classical and non-classical activation pathways. Its protective effects have been validated across multiple inflammation-related diseases (**Table 2**). It is noteworthy that MCC950 demonstrates significant potential in the prevention and treatment of skeletal muscle atrophy. MCC950 attenuates sepsis- and cancer cachexia-induced muscle atrophy through targeted inhibition of the NLRP3 inflammasome (113, 114), and alleviates LPS-induced myotube injury by suppressing oxidative stress and IL-1β-mediated inflammatory responses (115). In a Duchenne muscular dystrophy (DMD) model, MCC950 reduced pro-inflammatory factors and pyroptosis, enhanced muscle fibre size and maturation, and improved muscle function (116). Recent studies in diabetic muscle atrophy models revealed that MCC950 effectively inhibits NLRP3-mediated pyroptosis, improving muscle mass and motor function with synergistic effects when combined with aerobic exercise (64). These findings collectively indicate that targeting the NLRP3 inflammasome represents an effective strategy for preventing and treating skeletal muscle atrophy. Future research should elucidate the tissue-specific regulatory mechanisms of the pyroptosis pathway across different muscle atrophy models and develop more precise NLRP3 inhibitors or combined therapeutic strategies to advance the clinical translation for skeletal muscle atrophy and other pyroptosis-related diseases.

**Table 2:** Application of MCC950 in inflammation-related disease.

Research model/disease type	Targeted pathway/mechanism	Key findings/effects
Sepsis- and cancer cachexia- associated muscle atrophy	NLRP3 inflammasome inhibition	Attenuated muscle atrophy
Doxorubicin-induced myocardial injury	NLRP3 inflammasome inhibition	Attenuated myocardial injury
Myocardial hypoxia	Oxidative stress and pyroptosis	Alleviated oxidative stress and pyroptosis
Atherosclerosis	Macrophage pyroptosis and plaque progression	Inhibited macrophage pyroptosis and plaque progression
Cerebral ischemia-reperfusion	Astrocyte pyroptosis	Alleviated astrocyte pyroptosis
Epilepsy	TRPM7/STAT3/NLRP3 axis-mediated neuronal pyroptosis	Suppressed neuronal pyroptosis
Diabetes-associated periodontitis	Hyperglycemia-induced macrophage pyroptosis	Alleviated macrophage pyroptosis
Diethylbenzyl phthalate (DBP)-induced hepatic fibrosis	p38MAPK/NF-κB/NLRP3 pathway	Inhibited liver fibrosis

#### 4.3.2 OLT1177

OLT1177 (Dapansutrile) is an orally active, highly selective NLRP3 inflammasome inhibitor. It directly binds to the NACHT domain of NLRP3, thereby inhibiting inflammasome assembly and caspase-1 activation. This mechanism blocks the maturation and release of IL-1β and IL-18, regulates immune cell chemotaxis, and suppresses pyroptosis (117). Clinical studies indicate OLT1177 has therapeutic potential across multiple conditions, including gouty arthritis, cardiovascular disease, neurodegenerative disorders, inflammatory bowel disease, and periodontitis, with favourable safety profiles (117) (**Table 3**). In cardiovascular disease, it mitigates myocardial ischaemia-reperfusion injury, reduces infarct size, and improves cardiac function (118). Oral administration of OLT1177 enhances left ventricular ejection fraction and exercise tolerance in heart failure patients (119). Furthermore, in dengue-associated models, OLT1177 inhibits NLRP3 activation induced by viral EIII protein, alleviating neutrophil NETosis, platelet pyroptosis, and endothelial dysfunction (120–122). OLT1177 is also recognised as an NLRP3 inhibitor with therapeutic potential for acute lung injury (123). Notably, OLT1177 may be combined with other agents (such as lonafarnib or immune checkpoint inhibitors) to produce synergistic anti-inflammatory or anti-tumour effects (117).

**Table 3:** Application of OLT1177 in inflammation-related disease.

Research model/disease type	Targeted pathway/mechanism	Key findings/effects
LPS-Induced Systemic Inflammation	NLRP3 inflammasome; oxidative stress and IL-1β-mediated inflammatory responses	Reduces IL-1β levels in muscle tissue, alleviates oxidative stress, and reverses inflammatory metabolic wasting
Myocardial hypoxia	Inflammatory responses mediated by the NLRP3 inflammasome	Improves cardiac function; administration within 60 minutes of reperfusion can restore cardiac function
Gouty arthritis	Inhibition of the NLRP3 inflammasome	Inhibition of the maturation and release of IL-1β and IL-18
Heart failure	Inhibition of the NLRP3 inflammasome	Oral administration can improve left ventricular ejection fraction and exercise tolerance.
Dengue-associated endothelial dysfunction	Inhibition of the NLRP3 inflammasome	Alleviates neutrophil NETosis, platelet pyroptosis, and endothelial dysfunction
Acute lung injury	Inhibition of the NLRP3 inflammasome	Inhibit inflammation

Although direct studies on OLT1177 in skeletal muscle atrophy remain limited at present, its anti-inflammatory and cell-protective effects through inhibition of the NLRP3/pyroptosis pathway suggest it may serve as a potential therapeutic candidate for intervening in skeletal muscle wasting. Future research may further explore OLT1177 in muscle atrophy models (such as diabetic myopathy, cancer cachexia, and age-related sarcopenia), and evaluate its efficacy, either as monotherapy or in combination with exercise interventions, nutritional support, or other targeted therapies, in improving muscle mass and function whilst delaying atrophy progression. This approach may provide novel mechanistic strategies and translational pathways for the clinical prevention and treatment of skeletal muscle atrophy.

## 5 Conclusion and future perspectives

Pyroptosis, a programmed inflammatory death process mediated by Gasdermin proteins (centred on GSDMD), plays a pivotal role in skeletal muscle atrophy induced by multiple factors, including ageing, disease, disuse, and drug side effects. Its key mechanism relies on the widespread activation of the NLRP3 inflammasome/GSDMD axis. Once activated, this pathway directly induces inflammatory lysis and loss of muscle fibres. Concurrently, it releases inflammatory mediators, thereby establishing and amplifying a chronic inflammatory microenvironment. This further activates signalling pathways, including NF-κB, systemically promoting protein degradation whilst inhibiting synthesis, thereby exacerbating muscle wasting. Multi-tiered intervention strategies targeting this pathway, encompassing non-pharmacological approaches (such as exercise and specific nutrients), pharmacological agents (melatonin, BMP-7, trimethazidine, Dieckol), and highly selective NLRP3 inhibitors (MCC950 and OLT1177), have demonstrated preliminary promise in preclinical studies to alleviate muscle atrophy by inhibiting pyroptosis. Amongst these, specific inhibitors like MCC950 and OLT1177, alongside repurposed drug candidates such as trimethazidine, warrant particular attention. These compounds demonstrate anti-pyroptotic protective effects across multi-organ disease models and also offer novel drug targets and mechanistic insights for the prevention and treatment of skeletal muscle atrophy. However, in the process of advancing the clinical translation of the aforementioned intervention strategies, the clinical safety risks associated with long-term NLRP3 inhibition cannot be ignored. NLRP3 plays a critical role in host antimicrobial defence. Chronic systemic inhibition may impair anti-infection immunity, a concern particularly relevant for elderly sarcopenic patients and cancer cachexia patients who are already immunocompromised.

Beyond the safety concerns of individual inhibitors, the clinical translation of combined regimens integrating exercise, nutritional support, and pharmacological inhibitors faces substantial obstacles. Patients with sarcopenia and cachexia exhibit marked heterogeneity in etiology, inflammatory status, physical function, and nutritional condition, rendering standardised protocols difficult to formulate. Moreover, the optimal composition, sequencing, frequency, and duration of these multimodal interventions remain largely unexplored, and adherence to long-term structured regimens is notably low amongst elderly and cancer patients, further constraining feasibility. Regarding safety, concurrent or prolonged NLRP3 inhibitor use may potentiate immunosuppression and cumulative toxicity, warranting particular caution in frail populations. To address these barriers, feasible strategies include a tiered intervention model that reserves pharmacological agents for progressive or refractory cases whilst maintaining lifestyle modification as the foundation for early-stage atrophy, biomarker-guided patient stratification using circulating indicators such as IL-1β, IL-18, or GSDMD fragments, intermittent or sequential “lifestyle-first, drug-boost” protocols to balance efficacy with safety and adherence, and deployment of wearable devices and mobile health platforms for remote monitoring to facilitate dynamic adjustment and sustainable implementation of long-term combination regimens.

At the mechanistic level, future research should focus on: 1) elucidating the tissue-specific regulatory networks of pyroptosis in skeletal muscle, particularly the differential signalling patterns across distinct muscle fibre types and microenvironments to develop precise interventions. 2) Systematically deciphering the upstream signals (mitochondrial dysfunction, metabolic byproduct accumulation) that activate pyroptosis under various aetiologies at the mechanistic level, clarifying its interactions with apoptosis and autophagy. At the translational level, advance systematic validation of inhibitors such as MCC950 and OLT1177 in muscular atrophy disease models, whilst actively exploring synergistic effects with existing therapies, including exercise and nutritional support, to establish multimodal integrated treatment protocols. At the clinical implementation level, develop reliable biomarkers capable of dynamically reflecting muscle pyroptosis activity to enable patient stratification and treatment efficacy monitoring.

## References

[1] Duranti G . Special issue "Skeletal muscle adaptations to oxidative stress". Int J Mol Sci. (2025) 26(19):9809. doi: 10.3390/ijms26199809 41097073 PMC12525460

[2] Therdyothin A Prokopidis K Galli F Witard OC Isanejad M . The effects of omega-3 polyunsaturated fatty acids on muscle and whole-body protein synthesis: a systematic review and meta-analysis. Nutr Rev. (2025) 83:e131–43. doi: 10.1093/nutrit/nuae055 38777807 PMC11723138

[3] Maihemuti A Cui C Wang Q Amuti R Chau WW Chai S . Glucagon like peptide-1 receptor agonists for sarcopenia and muscle wasting disorders: a systematic review of efficacy and mechanisms. Aging Dis. (2025). doi: 10.14336/AD.2025.1165 41400575

[4] Troutman AD Arroyo E Sheridan EM D'Amico DJ Brandt PR Hinrichs R . Skeletal muscle atrophy in clinical and preclinical models of chronic kidney disease: a systematic review and meta-analysis. J Cachexia Sarcopenia Muscle. (2024) 15:21–35. doi: 10.1002/jcsm.13400 38062879 PMC10834351

[5] Segmen F Aydemir S Kayan T Bicer FTB Dogu C Aktekin EY . Clinical significance of sarcopenia defined by the cross-sectional area of the masseter muscle in cerebrovascular events: a retrospective cohort study. Medicina (Kaunas). (2025) 61(2):268. doi: 10.3390/medicina61020268 40005385 PMC11857208

[6] Bai Y Pan Y Liu X . Mechanistic insights into gasdermin-mediated pyroptosis. Nat Rev Mol Cell Biol. (2025) 26:501–21. doi: 10.1038/s41580-025-00837-0 40128620

[7] Huang C Li J Wu R Li Y Zhang C . Targeting pyroptosis for cancer immunotherapy: mechanistic insights and clinical perspectives. Mol Cancer. (2025) 24:131. doi: 10.1186/s12943-025-02344-4 40319304 PMC12049004

[8] Liu Y Pan R Ouyang Y Gu W Xiao T Yang H . Pyroptosis in health and disease: mechanisms, regulation and clinical perspective. Signal Transduct Target Ther. (2024) 9:245. doi: 10.1038/s41392-024-01958-2 39300122 PMC11413206

[9] Li Q Gao A Wu C Song X Liu W Cheng Y . The NLRP3 mediates masticatory muscle atrophy by pyroptosis and mitophagy. J Dent Res. (2025) 104:1556–66. doi: 10.1177/00220345251344295 40583159

[10] Berk BC Chavez CL George Hsu C . PDE10A inhibition reduces NLRP3 activation and pyroptosis in sepsis and nerve injury. Int J Mol Sci. (2025) 26(10):4498. doi: 10.3390/ijms26104498 40429643 PMC12111586

[11] Zhang S Guo S Wang P Song Y Yang L Sun Q . Dapagliflozin attenuates skeletal muscle atrophy in diabetic nephropathy mice through suppressing gasdermin D-mediated pyroptosis. Int Immunopharmacol. (2025) 148:114088. doi: 10.1016/j.intimp.2025.114088 39837016

[12] Tang X Zou Y Yang S Chen Z Zhou Z Peng X . Impaired mitophagy contributes to pyroptosis in sarcopenic obesity zebrafish skeletal muscle. Nutrients. (2025) 17(10):1711. doi: 10.3390/nu17101711 40431451 PMC12114099

[13] Wang L Jiao XF Wu C Li XQ Sun HX Shen XY . Trimetazidine attenuates dexamethasone-induced muscle atrophy via inhibiting NLRP3/GSDMD pathway-mediated pyroptosis. Cell Death Discov. (2021) 7:251. doi: 10.1038/s41420-021-00648-0 34537816 PMC8449784

[14] Behera J Ison J Voor MJ Tyagi N . Exercise-linked skeletal irisin ameliorates diabetes-associated osteoporosis by inhibiting the oxidative damage-dependent miR-150-FNDC5/pyroptosis axis. Diabetes. (2022) 71:2777–92. doi: 10.2337/db21-0573 35802043 PMC9750954

[15] Shi J Zhao Y Wang Y Gao W Ding J Li P . Inflammatory caspases are innate immune receptors for intracellular LPS. Nature. (2014) 514:187–92. doi: 10.1038/nature13683 25119034

[16] Shi J Zhao Y Wang K Shi X Wang Y Huang H . Cleavage of GSDMD by inflammatory caspases determines pyroptotic cell death. Nature. (2015) 526:660–5. doi: 10.1038/nature15514 26375003

[17] Wang Y Gao W Shi X Ding J Liu W He H . Chemotherapy drugs induce pyroptosis through caspase-3 cleavage of a gasdermin. Nature. (2017) 547:99–103. doi: 10.1038/nature22393 28459430

[18] Liu Y Fang Y Chen X Wang Z Liang X Zhang T . Gasdermin E-mediated target cell pyroptosis by CAR T cells triggers cytokine release syndrome. Sci Immunol. (2020) 5(43):eaax7969. doi: 10.1126/sciimmunol.aax7969 31953257

[19] Rao Z Zhu Y Yang P Chen Z Xia Y Qiao C . Pyroptosis in inflammatory diseases and cancer. Theranostics. (2022) 12:4310–29. doi: 10.7150/thno.71086 35673561 PMC9169370

[20] Zou J Zheng Y Huang Y Tang D Kang R Chen R . The versatile gasdermin family: their function and roles in diseases. Front Immunol. (2021) 12:751533. doi: 10.3389/fimmu.2021.751533 34858408 PMC8632255

[21] Hsu SK Chen YE Shu ED Ko CC Chang WT Lin IL . The pyroptotic and nonpyroptotic roles of gasdermins in modulating cancer progression and their perspectives on cancer therapeutics. Arch Immunol Ther Exp (Warsz). (2023) 71:14. doi: 10.1007/s00005-023-00678-9 37258998

[22] Zhu C Xu S Jiang R Yu Y Bian J Zou Z . The gasdermin family: emerging therapeutic targets in diseases. Signal Transduct Target Ther. (2024) 9:87. doi: 10.1038/s41392-024-01801-8 38584157 PMC10999458

[23] Dubyak GR Miller BA Pearlman E . Pyroptosis in neutrophils: multimodal integration of inflammasome and regulated cell death signaling pathways. Immunol Rev. (2023) 314:229–49. doi: 10.1111/imr.13186 36656082 PMC10407921

[24] Ni FX Wang HX Chen PS Huang HJ Chen HH Huang DH . Pyroptosis-immune cell cross talk in asthma: from molecular mechanisms to precision therapeutics. Front Immunol. (2025) 16:1727122. doi: 10.3389/fimmu.2025.1727122 41394843 PMC12698424

[25] Zhang CC Li CG Wang YF Xu LH He XH Zeng QZ . Chemotherapeutic paclitaxel and cisplatin differentially induce pyroptosis in A549 lung cancer cells via caspase-3/GSDME activation. Apoptosis. (2019) 24:312–25. doi: 10.1007/s10495-019-01515-1 30710195

[26] Yu P Zhang X Liu N Tang L Peng C Chen X . Pyroptosis: mechanisms and diseases. Signal Transduct Target Ther. (2021) 6:128. doi: 10.1038/s41392-021-00507-5 33776057 PMC8005494

[27] Pandey A Li Z Gautam M Ghosh A Man SM . Molecular mechanisms of emerging inflammasome complexes and their activation and signaling in inflammation and pyroptosis. Immunol Rev. (2025) 329:e13406. doi: 10.1111/imr.13406 39351983 PMC11742652

[28] Wei X Xie F Zhou X Wu Y Yan H Liu T . Role of pyroptosis in inflammation and cancer. Cell Mol Immunol. (2022) 19:971–92. doi: 10.1038/s41423-022-00905-x 35970871 PMC9376585

[29] Liu Y Bi X Zhang Y Wang Y Ding W . Mitochondrial dysfunction/NLRP3 inflammasome axis contributes to angiotensin II-induced skeletal muscle wasting via PPAR-gamma. Lab Invest. (2020) 100:712–26. doi: 10.1038/s41374-019-0355-1 31857693

[30] Li T Zhao X Xu Z Yang F Li Z Bai X . Selenomethionine attenuates sepsis-induced skeletal muscle atrophy by inhibiting ROS/NLRP3 signaling. Shock. (2026) 65:262–74. doi: 10.1097/SHK.0000000000002690 40961381

[31] Fang WY Tseng YT Lee TY Fu YC Chang WH Lo WW . Triptolide prevents LPS-induced skeletal muscle atrophy via inhibiting NF-kappaB/TNF-alpha and regulating protein synthesis/degradation pathway. Br J Pharmacol. (2021) 178:2998–3016. doi: 10.1111/bph.15472 33788266

[32] Chen Y Chen R Ji X Zeng Z Guan C . NLRP3 inflammasome-mediated pyroptosis in diabetic nephropathy: pathogenic mechanisms and therapeutic targets. J Inflammation Res. (2025) 18:8399–418. doi: 10.2147/JIR.S524246 40585041 PMC12206412

[33] Xu J Zhang L Duan Y Sun F Odeh N He Y . NEK7 phosphorylation amplifies NLRP3 inflammasome activation downstream of potassium efflux and gasdermin D. Sci Immunol. (2025) 10:eadl2993. doi: 10.1126/sciimmunol.adl2993 39752537 PMC12020992

[34] Yin F Zheng PQ Zhao LQ Wang YZ Miao NJ Zhou ZL . Caspase-11 promotes NLRP3 inflammasome activation via the cleavage of pannexin1 in acute kidney disease. Acta Pharmacol Sin. (2022) 43:86–95. doi: 10.1038/s41401-021-00619-2 33758356 PMC8724289

[35] Zhai Z Yang F Xu W Han J Luo G Li Y . Attenuation of rheumatoid arthritis through the inhibition of tumor necrosis factor-induced caspase 3/gasdermin E-mediated pyroptosis. Arthritis Rheumatol. (2022) 74:427–40. doi: 10.1002/art.41963 34480835 PMC9305212

[36] Shen X Wang H Weng C Jiang H Chen J . Caspase 3/GSDME-dependent pyroptosis contributes to chemotherapy drug-induced nephrotoxicity. Cell Death Dis. (2021) 12:186. doi: 10.1038/s41419-021-03458-5 33589596 PMC7884686

[37] Orning P Weng D Starheim K Ratner D Best Z Lee B . Pathogen blockade of TAK1 triggers caspase-8-dependent cleavage of gasdermin D and cell death. Science. (2018) 362:1064–9. doi: 10.1126/science.aau2818 30361383 PMC6522129

[38] Chao KL Kulakova L Herzberg O . Gene polymorphism linked to increased asthma and IBD risk alters gasdermin-B structure, a sulfatide and phosphoinositide binding protein. Proc Natl Acad Sci USA. (2017) 114:E1128–37. doi: 10.1073/pnas.1616783114 28154144 PMC5321033

[39] Zhou Z He H Wang K Shi X Wang Y Su Y . Granzyme A from cytotoxic lymphocytes cleaves GSDMB to trigger pyroptosis in target cells. Science. (2020) 368(6494):eaaz7548. doi: 10.1126/science.aaz7548 32299851

[40] Zhao Q Chen DP Chen HD Wang YZ Shi W Lu YT . NK-cell-elicited gasdermin-D-dependent hepatocyte pyroptosis induces neutrophil extracellular traps that facilitate HBV-related acute-on-chronic liver failure. Hepatology. (2025) 81:917–31. doi: 10.1097/HEP.0000000000000868 38537134

[41] Zhang Z Zhang Y Xia S Kong Q Li S Liu X . Gasdermin E suppresses tumour growth by activating anti-tumour immunity. Nature. (2020) 579:415–20. doi: 10.1038/s41586-020-2071-9 32188940 PMC7123794

[42] Sayer AA Cooper R Arai H Cawthon PM Ntsama Essomba MJ Fielding RA . Sarcopenia. Nat Rev Dis Primers. (2024) 10:68. doi: 10.1038/s41572-024-00550-w 39300120

[43] Liu D Wang S Liu S Wang Q Che X Wu G . Frontiers in sarcopenia: advancements in diagnostics, molecular mechanisms, and therapeutic strategies. Mol Aspects Med. (2024) 97:101270. doi: 10.1016/j.mam.2024.101270 38583268

[44] Peng J Zou M Zhang Q Liu D Chen S Fang R . Symphony of regulated cell death: unveiling therapeutic horizons in sarcopenia. Metabolism. (2025) 172:156359. doi: 10.1016/j.metabol.2025.156359 40752569

[45] Wu J Lin S Chen W Lian G Wu W Chen A . TNF-alpha contributes to sarcopenia through caspase-8/caspase-3/GSDME-mediated pyroptosis. Cell Death Discov. (2023) 9:76. doi: 10.1038/s41420-023-01365-6 36823174 PMC9950087

[46] Li CW Yu K Shyh-Chang N Jiang Z Liu T Ma S . Pathogenesis of sarcopenia and the relationship with fat mass: descriptive review. J Cachexia Sarcopenia Muscle. (2022) 13:781–94. doi: 10.1002/jcsm.12901 35106971 PMC8977978

[47] Fu P Gong L Yang L Tang S Ma F . Weight bearing training alleviates muscle atrophy and pyroptosis of middle-aged rats. Front Endocrinol (Lausanne). (2023) 14:1202686. doi: 10.3389/fendo.2023.1202686 37720530 PMC10499618

[48] Sayed RKA Fernandez-Ortiz M Diaz-Casado ME Aranda-Martinez P Fernandez-Martinez J Guerra-Librero A . Lack of NLRP3 inflammasome activation reduces age-dependent sarcopenia and mitochondrial dysfunction, favoring the prophylactic effect of melatonin. J Gerontol A Biol Sci Med Sci. (2019) 74:1699–708. doi: 10.1093/gerona/glz079 30869745

[49] Sayed RKA Fernandez-Ortiz M Diaz-Casado ME Rusanova I Rahim I Escames G . The protective effect of melatonin against age-associated, sarcopenia-dependent tubular aggregate formation, lactate depletion, and mitochondrial changes. J Gerontol A Biol Sci Med Sci. (2018) 73:1330–8. doi: 10.1093/gerona/gly059 29562315

[50] Wang F Liang Q Ma Y Sun M Li T Lin L . Silica nanoparticles induce pyroptosis and cardiac hypertrophy via ROS/NLRP3/caspase-1 pathway. Free Radic Biol Med. (2022) 182:171–81. doi: 10.1016/j.freeradbiomed.2022.02.027 35219847

[51] Xu H Gao Z Yao X Sun J Chen B . The role of cell death in the physiological and pathological processes of skeletal muscle. Front Physiol. (2025) 16:1717233. doi: 10.3389/fphys.2025.1717233 41334559 PMC12665578

[52] Cao Q Liu J Huang G Wang SY Lu GD Huang Y . GSDME-IL-18 pyroptotic axis prevents myosteatosis by expanding tissue-resident macrophages to promote muscle regeneration. J Clin Invest. (2026) 136(8):e198076. doi: 10.1172/JCI198076 41701674 PMC13078871

[53] Ogura M Matsuoka H Shinohara S Umeki Y Mastumoto N Mizuno T . The optimal timing for initiating anamorelin in the treatment of cancer cachexia. Cureus. (2025) 17:e81622. doi: 10.7759/cureus.81622 40330331 PMC12051079

[54] Setiawan T Sari IN Wijaya YT Julianto NM Muhammad JA Lee H . Cancer cachexia: molecular mechanisms and treatment strategies. J Hematol Oncol. (2023) 16:54. doi: 10.1186/s13045-023-01454-0 37217930 PMC10204324

[55] Babic A Rosenthal MH Sundaresan TK Khalaf N Lee V Brais LK . Adipose tissue and skeletal muscle wasting precede clinical diagnosis of pancreatic cancer. Nat Commun. (2023) 14:4317. doi: 10.1038/s41467-023-40024-3 37463915 PMC10354105

[56] Qi H Zhang X Zhang Z Gao Y Tian D Zhao G . The extract of chrysanthemum flos mitigates post-stroke sarcopenia by inhibiting PANoptosis and restoring muscle homeostasis. Phytomedicine. (2025) 142:156784. doi: 10.1016/j.phymed.2025.156784 40311590

[57] Zhou T Wang S Pan Y Dong X Wu L Meng J . Irisin ameliorated skeletal muscle atrophy by inhibiting fatty acid oxidation and pyroptosis induced by palmitic acid in chronic kidney disease. Kidney Blood Press Res. (2023) 48:628–41. doi: 10.1159/000533926 37717561 PMC10614467

[58] Kim HG Huot JR Pin F Belcher DJ Bonetto A Nader GA . Metastatic or xenograft colorectal cancer models induce divergent anabolic deficits and expression of pro-inflammatory effectors of muscle wasting in a tumor-type-dependent manner. J Appl Physiol (1985). (2022) 133:1273–83. doi: 10.1152/japplphysiol.00247.2022 36201323 PMC9678410

[59] Huang N Kny M Riediger F Busch K Schmidt S Luft FC . Deletion of Nlrp3 protects from inflammation-induced skeletal muscle atrophy. Intensive Care Med Exp. (2017) 5:3. doi: 10.1186/s40635-016-0115-0 28097512 PMC5241267

[60] You Z Huang X Xiang Y Dai J Xu L Jiang J . Ablation of NLRP3 inflammasome attenuates muscle atrophy via inhibiting pyroptosis, proteolysis and apoptosis following denervation. Theranostics. (2023) 13:374–90. doi: 10.7150/thno.74831 36593964 PMC9800723

[61] Li S Wu L Xie J Zhou G Wen X Deng L . Edaravone improves motor dysfunction following brachial plexus avulsion injury in rats. ACS Chem Neurosci. (2025) 16:479–89. doi: 10.1021/acschemneuro.4c00717 39791183

[62] Dezfouli MA Shalilahmadi D Shamsaei G Esmaeili A Majdinasab N Rashidi SK . Circulating miR-223/NLRP3 axis and IL-1beta level in functional disease progression of amyotrophic lateral sclerosis. Acta Neurol Belg. (2025) 125:783–91. doi: 10.1007/s13760-025-02764-5 40097890

[63] Yang J Wang M Shi L Fang X Gao C Ma L . The stimulator of interferon genes deficiency attenuates diabetic myopathy through inhibiting NLRP3-mediated pyroptosis. J Cachexia Sarcopenia Muscle. (2025) 16:e13649. doi: 10.1002/jcsm.13649 39602084 PMC11670168

[64] Yan X Fu P Zhang Y Ling D Reynolds L Hua W . MCC950 ameliorates diabetic muscle atrophy in mice by inhibition of pyroptosis and its synergistic effect with aerobic exercise. Molecules. (2024) 29(3):712. doi: 10.3390/molecules29030712 38338456 PMC10856337

[65] Aluganti Narasimhulu C Singla DK . Amelioration of diabetes-induced inflammation mediated pyroptosis, sarcopenia, and adverse muscle remodelling by bone morphogenetic protein-7. J Cachexia Sarcopenia Muscle. (2021) 12:403–20. doi: 10.1002/jcsm.12662 33463042 PMC8061343

[66] Zhang Y Li T Liu Y Wang C Wang D Xu L . Gsdmd knockout alleviates sepsis-associated skeletal muscle atrophy by inhibiting Il18/Ampk signaling. Shock. (2024) 62:565–73. doi: 10.1097/SHK.0000000000002430 39227368

[67] Ding J Li F Cong Y Miao J Wu D Liu B . Trichostatin A inhibits skeletal muscle atrophy induced by cigarette smoke exposure in mice. Life Sci. (2019) 235:116800. doi: 10.1016/j.lfs.2019.116800 31472151

[68] Bae S Mai VH Mun S Dong D Han K Park S . Lonafarnib protects against muscle atrophy induced by dexamethasone. J Cachexia Sarcopenia Muscle. (2025) 16:e13665. doi: 10.1002/jcsm.13665 39686867 PMC11696026

[69] Mishra S Cosentino C Tamta AK Khan D Srinivasan S Ravi V . Sirtuin 6 inhibition protects against glucocorticoid-induced skeletal muscle atrophy by regulating IGF/PI3K/AKT signaling. Nat Commun. (2022) 13:5415. doi: 10.1038/s41467-022-32905-w 36109503 PMC9478160

[70] Park E Choi H Truong CS Jun HS . The inhibition of autophagy and pyroptosis by an ethanol extract of Nelumbo nucifera leaf contributes to the amelioration of dexamethasone-induced muscle atrophy. Nutrients. (2023) 15(4):804. doi: 10.3390/nu15040804 36839161 PMC9965294

[71] Dang R Hou X Huang X Huang C Zhao X Wang X . Effects of the glucocorticoid-mediated mitochondrial translocation of glucocorticoid receptors on oxidative stress and pyroptosis in BV-2 microglia. J Mol Neurosci. (2024) 74:30. doi: 10.1007/s12031-024-02192-9 38478195

[72] Oh S Yang J Park C Son K Byun K . Dieckol attenuated glucocorticoid-induced muscle atrophy by decreasing NLRP3 inflammasome and pyroptosis. Int J Mol Sci. (2021) 22(15):8057. doi: 10.3390/ijms22158057 34360821 PMC8348567

[73] Xie W Peng M Liu Y Zhang B Yi L Long Y . Simvastatin induces pyroptosis via ROS/caspase-1/GSDMD pathway in colon cancer. Cell Commun Signal. (2023) 21:329. doi: 10.1186/s12964-023-01359-y 37974278 PMC10652480

[74] Zhu PF Wang MX Chen ZL Yang L . Targeting the tumor microenvironment: a literature review of the novel anti-tumor mechanism of statins. Front Oncol. (2021) 11:761107. doi: 10.3389/fonc.2021.761107 34858839 PMC8632059

[75] Chen ZL Guo C Zou YY Feng C Yang DX Sun CC . Aerobic exercise enhances mitochondrial homeostasis to counteract D-galactose-induced sarcopenia in zebrafish. Exp Gerontol. (2023) 180:112265. doi: 10.1016/j.exger.2023.112265 37482108

[76] Yang X Wu Y Zhang M Zhang L Zhao T Qian W . Piceatannol protects against age-related hearing loss by inhibiting cellular pyroptosis and inflammation through regulated Caspase11-GSDMD pathway. BioMed Pharmacother. (2023) 163:114704. doi: 10.1016/j.biopha.2023.114704 37100013

[77] Matikainen S Nyman TA Cypryk W . Function and regulation of noncanonical caspase-4/5/11 inflammasome. J Immunol. (2020) 204:3063–9. doi: 10.4049/jimmunol.2000373 32513874

[78] Wang C Liu Y Zhang Y Wang D Xu L Li Z . Targeting NAT10 protects against sepsis-induced skeletal muscle atrophy by inhibiting ROS/NLRP3. Life Sci. (2023) 330:121948. doi: 10.1016/j.lfs.2023.121948 37467885

[79] McBride MJ Foley KP D'Souza DM Li YE Lau TC Hawke TJ . The NLRP3 inflammasome contributes to sarcopenia and lower muscle glycolytic potential in old mice. Am J Physiol Endocrinol Metab. (2017) 313:E222–32. doi: 10.1152/ajpendo.00060.2017 28536183 PMC5582883

[80] Pansarasa O Felzani G Vecchiet J Marzatico F . Antioxidant pathways in human aged skeletal muscle: relationship with the distribution of type II fibers. Exp Gerontol. (2002) 37:1069–75. doi: 10.1016/s0531-5565(02)00085-2 12213557

[81] Gueguen N Lefaucheur L Fillaut M Herpin P . Muscle fiber contractile type influences the regulation of mitochondrial function. Mol Cell Biochem. (2005) 276:15–20. doi: 10.1007/s11010-005-2464-y 16132680

[82] Morales-Scholz MG Wette SG Stokie JR Tepper BT Swinton C Hamilton DL . Muscle fiber type-specific autophagy responses following an overnight fast and mixed meal ingestion in human skeletal muscle. Am J Physiol Endocrinol Metab. (2022) 323:E242–53. doi: 10.1152/ajpendo.00015.2022 35793481

[83] Bailleul C Hodson N Abou Sawan S Kumbhare D Moore DR Gillen JB . The influence of sex on fiber-specific indices of oxidative capacity in human skeletal muscle. Am J Physiol Regul Integr Comp Physiol. (2025) 329:R70–80. doi: 10.1152/ajpregu.00298.2024 40408260

[84] Zhao Y He X Yang X Hong Z Xu Y Xu J . CircFndc3b mediates exercise-induced neuroprotection by mitigating microglial/macrophage pyroptosis via the ENO1/KLF2 axis in stroke mice. Adv Sci (Weinh). (2025) 12:e2403818. doi: 10.1002/advs.202403818 39467260 PMC11714177

[85] Liu J Jia S Yang Y Piao L Wang Z Jin Z . Exercise induced meteorin-like protects chondrocytes against inflammation and pyroptosis in osteoarthritis by inhibiting PI3K/Akt/NF-kappaB and NLRP3/caspase-1/GSDMD signaling. BioMed Pharmacother. (2023) 158:114118. doi: 10.1016/j.biopha.2022.114118 36527845

[86] Li Y Wang X Meng X Xia C Yang C Wang J . Aerobic exercise inhibits GSDME-dependent myocardial cell pyroptosis to protect ischemia-reperfusion injury. Mol Med. (2024) 30:273. doi: 10.1186/s10020-024-01048-7 39719560 PMC11668014

[87] Yang B Xu J Dao X Huang Y Liang J Huang J . Aerobic exercise and PI3K inhibitor ameliorate obesity cardiomyopathy by alleviating pyroptosis in middle-aged mice. Int J Mol Sci. (2025) 26(10):4935. doi: 10.3390/ijms26104935 40430076 PMC12112324

[88] Liu Z Yang Y Song L Ruan X Li X He Y . Potential role of aerobic exercise in attenuating diabetic cardiomyopathy via modulation of P2X4-mediated NLRP3 inflammasome activation and pyroptosis. J Innate Immun. (2025) 17:539–65. doi: 10.1159/000548603 41036663 PMC12659644

[89] Ganapathy A Nieves JW . Nutrition and sarcopenia—what do we know?. Nutrients. (2020) 12(6):1755. doi: 10.3390/nu12061755 32545408 PMC7353446

[90] Smith GI Julliand S Reeds DN Sinacore DR Klein S Mittendorfer B . Fish oil-derived n-3 PUFA therapy increases muscle mass and function in healthy older adults. Am J Clin Nutr. (2015) 102:115–22. doi: 10.3945/ajcn.114.105833 25994567 PMC4480667

[91] Calder PC . Omega-3 fatty acids and inflammatory processes: from molecules to man. Biochem Soc Trans. (2017) 45:1105–15. doi: 10.1042/BST20160474 28900017

[92] Xu D Lu Y Yang X Pan D Wang Y Yin S . Effects of fish oil-derived n-3 polyunsaturated fatty acid on body composition, muscle strength and physical performance in older people: a secondary analysis of a randomised, double-blind, placebo-controlled trial. Age Ageing. (2022) 51(12):afac274. doi: 10.1093/ageing/afac274 36571774

[93] Yu Z Chen N Xu H . Short-chain fatty acids alleviate sarcopenia in rats via modulation of the NF-kappaB signaling pathway. Sci Rep. (2026) 16(1):16167. doi: 10.1038/s41598-026-47873-0 41957413 PMC13201837

[94] Remelli F Vitali A Zurlo A Volpato S . Vitamin D deficiency and sarcopenia in older persons. Nutrients. (2019) 11(12):2861. doi: 10.3390/nu11122861 31766576 PMC6950416

[95] Garcia LA King KK Ferrini MG Norris KC Artaza JN . 1,25(OH)_2_vitamin D_3_ stimulates myogenic differentiation by inhibiting cell proliferation and modulating the expression of promyogenic growth factors and myostatin in C2C12 skeletal muscle cells. Endocrinology. (2011) 152:2976–86. doi: 10.1210/en.2011-0159 21673099 PMC3138228

[96] Liu D Zhang Z Wang Y Liang X Chang Y Wang C . Rhodiola rosea-derived exosome-like nanovesicles inhibit vascular endothelial pyroptosis in the treatment of limb skeletal muscle ischemic injury through the TXNIP/NLNP3 pathway. Regener Biomater. (2025) 12:rbaf113. doi: 10.1093/rb/rbaf113 41357849 PMC12681252

[97] Park Y Choi JE Hwang HS . Protein supplementation improves muscle mass and physical performance in undernourished prefrail and frail elderly subjects: a randomized, double-blind, placebo-controlled trial. Am J Clin Nutr. (2018) 108:1026–33. doi: 10.1093/ajcn/nqy214 30475969

[98] Maity J Dey TD Banerjee A Chattopadhyay A Das AR Bandyopadhyay D . Melatonin ameliorates myocardial infarction in obese diabetic individuals: the possible involvement of macrophage apoptotic factors. J Pineal Res. (2023) 74:e12847. doi: 10.1111/jpi.12847 36456538

[99] Zhang W Wang X Tang Y Huang C . Melatonin alleviates doxorubicin-induced cardiotoxicity via inhibiting oxidative stress, pyroptosis and apoptosis by activating Sirt1/Nrf2 pathway. BioMed Pharmacother. (2023) 162:114591. doi: 10.1016/j.biopha.2023.114591 36965257

[100] Chen R Yang M . Melatonin inhibits OGD/R-induced H9c2 cardiomyocyte pyroptosis via regulation of MT2/miR-155/FOXO3a/ARC axis. Int Heart J. (2022) 63:327–37. doi: 10.1536/ihj.21-571 35354753

[101] Li Q Feng L Tian Y Guo E Li Y Niu J . Melatonin alleviates neuroinflammation in ischemic stroke by regulating cGAS-mediated microglial pyroptosis signaling. Neural Regen Res. (2026) 21(6):2380–8. doi: 10.4103/NRR.NRR-D-24-01070 PMC13211821 40536992

[102] Gao Z Luo K Hu Y Niu Y Zhu X Li S . Melatonin alleviates chronic stress-induced hippocampal microglia pyroptosis and subsequent depression-like behaviors by inhibiting Cathepsin B/NLRP3 signaling pathway in rats. Transl Psychiatry. (2024) 14:166. doi: 10.1038/s41398-024-02887-y 38538614 PMC10973390

[103] Wang X Wang W Zhang R Ma B Ni L Feng H . Melatonin attenuates high glucose-induced endothelial cell pyroptosis by activating the Nrf2 pathway to inhibit NLRP3 inflammasome activation. Mol Med Rep. (2023) 27(3):71. doi: 10.3892/mmr.2023.12958 36799176 PMC9942260

[104] Cong L Liu X Bai Y Qin Q Zhao L Shi Y . Melatonin alleviates pyroptosis by regulating the SIRT3/FOXO3alpha/ROS axis and interacting with apoptosis in atherosclerosis progression. Biol Res. (2023) 56:62. doi: 10.1186/s40659-023-00479-6 38041171 PMC10693060

[105] Chen X Kadier M Shi M Li K Chen H Xia Y . Targeting melatonin to mitochondria mitigates castration-resistant prostate cancer by inducing pyroptosis. Small. (2025) 21:e2408996. doi: 10.1002/smll.202408996 40285589

[106] Narasimhulu CA Singla DK . BMP-7 attenuates sarcopenia and adverse muscle remodeling in diabetic mice via alleviation of lipids, inflammation, HMGB1, and pyroptosis. Antioxidants (Basel). (2023) 12(2):331. doi: 10.3390/antiox12020331 36829889 PMC9952667

[107] Elmadbouh I Singla DK . BMP-7 attenuates inflammation-induced pyroptosis and improves cardiac repair in diabetic cardiomyopathy. Cells. (2021) 10. doi: 10.3390/cells10102640 PMC8533936 34685620

[108] Yu XJ Wang YG Lu R Guo XZ Qu YK Wang SX . BMP7 ameliorates intervertebral disc degeneration in type 1 diabetic rats by inhibiting pyroptosis of nucleus pulposus cells and NLRP3 inflammasome activity. Mol Med. (2023) 29:30. doi: 10.1186/s10020-023-00623-8 36858954 PMC9979491

[109] Chen X Lin S Dai S Han J Shan P Wang W . Trimetazidine affects pyroptosis by targeting GSDMD in myocardial ischemia/reperfusion injury. Inflammation Res. (2022) 71:227–41. doi: 10.1007/s00011-021-01530-6 34993560

[110] Chen J Wang B Lai J Braunstein Z He M Ruan G . Trimetazidine attenuates cardiac dysfunction in endotoxemia and sepsis by promoting neutrophil migration. Front Immunol. (2018) 9:2015. doi: 10.3389/fimmu.2018.02015 30233596 PMC6131494

[111] Gu Y Liu M Liu Y Zhang M Liang W Cui S . Novel chiral borneol derivatives as potential cardioprotective agents: Design, synthesis and biological evaluation. Bioorg Chem. (2025) 163:108767. doi: 10.1016/j.bioorg.2025.108767 40712226

[112] Oh S Son M Byun KA Jang JT Choi CH Son KH . Attenuating effects of dieckol on high-fat diet-induced nonalcoholic fatty liver disease by decreasing the NLRP3 inflammasome and pyroptosis. Mar Drugs. (2021) 19(6):318. doi: 10.3390/md19060318 34070893 PMC8227003

[113] Liu Y Wang K Xu Z Zhao X Li Z Bai X . MCC950-loaded M12-liposome nanoparticles for targeted inhibition of NLRP3 inflammasome in sepsis-induced muscle atrophy. J Cachexia Sarcopenia Muscle. (2026) 17:e70285. doi: 10.1002/jcsm.70285 41983452 PMC13080895

[114] Pan X Fu J Gu X Fan M Pan L Zhao Y . Inhibiting the NLRP3 inflammasome with MCC950 ameliorates muscle atrophy in cancer cachexia. Eur J Pharmacol. (2026) 1017:178654. doi: 10.1016/j.ejphar.2026.178654 41679687

[115] Eggelbusch M Shi A Broeksma BC Vazquez-Cruz M Soares MN de Wit GMJ . The NLRP3 inflammasome contributes to inflammation-induced morphological and metabolic alterations in skeletal muscle. J Cachexia Sarcopenia Muscle. (2022) 13:3048–61. doi: 10.1002/jcsm.13062 35978267 PMC9745466

[116] Dubuisson N Davis-Lopez de Carrizosa MA Versele R Selvais CM Noel L Van den Bergh PYD . Inhibiting the inflammasome with MCC950 counteracts muscle pyroptosis and improves Duchenne muscular dystrophy. Front Immunol. (2022) 13:1049076. doi: 10.3389/fimmu.2022.1049076 36569900 PMC9770793

[117] Bai F Wang D Wu Y . Dapansutrile in multidisciplinary therapeutic applications: mechanisms and clinical perspectives. Front Pharmacol. (2025) 16:1731165. doi: 10.3389/fphar.2025.1731165 41415576 PMC12708538

[118] Toldo S Mauro AG Cutter Z Van Tassell BW Mezzaroma E Del Buono MG . The NLRP3 inflammasome inhibitor, OLT1177 (Dapansutrile), reduces infarct size and preserves contractile function after ischemia reperfusion injury in the mouse. J Cardiovasc Pharmacol. (2019) 73:215–22. doi: 10.1097/FJC.0000000000000658 30747785

[119] Wohlford GF Van Tassell BW Billingsley HE Kadariya D Canada JM Carbone S . Phase 1B, randomized, double-blinded, dose escalation, single-center, repeat dose safety and pharmacodynamics study of the oral NLRP3 inhibitor dapansutrile in subjects with NYHA II-III systolic heart failure. J Cardiovasc Pharmacol. (2020) 77:49–60. doi: 10.1097/FJC.0000000000000931 33235030 PMC7774821

[120] Lien TS Sun DS Hung SC Wu WS Chang HH . Dengue virus envelope protein domain III induces Nlrp3 inflammasome-dependent NETosis-mediated inflammation in mice. Front Immunol. (2021) 12:618577. doi: 10.3389/fimmu.2021.618577 33815373 PMC8009969

[121] Lien TS Chan H Sun DS Wu JC Lin YY Lin GL . Exposure of platelets to dengue virus and envelope protein domain III induces Nlrp3 inflammasome-dependent platelet cell death and thrombocytopenia in mice. Front Immunol. (2021) 12:616394. doi: 10.3389/fimmu.2021.616394 33995345 PMC8118162

[122] Lien TS Sun DS Wu CY Chang HH . Exposure to dengue envelope protein domain III induces Nlrp3 inflammasome-dependent endothelial dysfunction and hemorrhage in mice. Front Immunol. (2021) 12:617251. doi: 10.3389/fimmu.2021.617251 33717109 PMC7947687

[123] Wang J Li LL Zhao ZA Niu CY Zhao ZG . NLRP3 inflammasome-mediated pyroptosis in acute lung injury: Roles of main lung cell types and therapeutic perspectives. Int Immunopharmacol. (2025) 154:114560. doi: 10.1016/j.intimp.2025.114560 40184810

